# FOXP3^+^ Treg Cells and Gender Bias in Autoimmune Diseases

**DOI:** 10.3389/fimmu.2015.00493

**Published:** 2015-09-28

**Authors:** Jia Nie, Yang Yang Li, Song Guo Zheng, Andy Tsun, Bin Li

**Affiliations:** ^1^Key Laboratory of Molecular Virology and Immunology, Institut Pasteur of Shanghai, Shanghai Institutes for Biological Sciences, Chinese Academy of Sciences, Shanghai, China; ^2^Clinical Immunology Center, The Third Affiliated Hospital, Sun Yat-Sen University, Guangzhou, China; ^3^Department of Medicine, Division of Rheumatology, Penn State Hershey College of Medicine, Hershey, PA, USA; ^4^Innovent Biologics Inc., Suzhou, China

**Keywords:** Treg cells, FOXP3, gender, autoimmunity, inflammation

## Abstract

CD4^+^CD25^+^ regulatory T (Treg) cells play a pivotal role in the maintenance of immune homeostasis, where the X-linked master transcription factor forkhead box P3 (FOXP3) determines Treg cell development and function. Genetic deficiency of *foxp3* induces dysfunction of Treg cells and immuno-dysregulation, polyendocrinopathy, enteropathy, and X-linked syndrome in humans. Functionally deficient Treg cells or the development of exTreg cells positively correlate with autoimmune diseases, such as systemic lupus erythematosus (SLE), multiple sclerosis (MS), and ankylosing spondylitis (AS). In general, females are more susceptible to SLE and MS but less susceptible to AS, where the expression of FOXP3 and its protein complex are perturbed by multiple factors, including hormonal fluctuations, inflammatory cytokines, and danger signals. Therefore, it is critical to explore the potential molecular mechanisms involved and these differences linked to gender. Here, we review recent findings on the regulation of FOXP3 activity in Treg cells and also discuss gender difference in the determination of Treg cell function in autoimmune diseases.

## Introduction

Regulatory T (Treg) cells, via their immune suppressive capability, play an indispensable role in maintaining immune homeostasis and preventing autoimmunity induced by excessive, misdirected, or unnecessary immune activation. Surface-expressed cytotoxic T lymphocyte-associated antigen 4 (CTLA-4) mediates suppression of target cells by cell–cell contact (1–4). Treg cells can also reduce T cell activation and proliferation through CD39–CD73-mediated production of metabolic adenosine (5). Meanwhile, Treg cells have been shown to harbor cytotoxic capacity and induce target cell apoptosis through release of granzymes A/B and perforin (4). Anti-inflammatory cytokines that are secreted by Treg cells can also induce immune tolerance (6, 7).

Under pathogenic conditions, such as systemic lupus erythematosus (SLE) and multiple sclerosis (MS), Treg cells exhibit plasticity to some extent and may mimic T helper-like phenotypes. Recent studies have provided insight into the understanding of the stability and activity of forkhead box P3 (FOXP3) in Treg cells regulated by T cell receptor (TCR) signaling, inflammatory cytokines, and danger signals. Here, we discuss the cellular and molecular mechanisms underlying FOXP3-mediated regulation of Treg cells and also the possible effect that gender difference has on Treg cells and autoimmune diseases.

## FOXP3 Mutations and Autoimmunity

The transcription factor FOXP3 belongs to the fork-winged helix family and is encoded by the *foxp3* gene on the X chromosome. Genetic deletion of the *foxp3* gene and the loss of Treg cells promote the development of autoimmune and inflammatory syndromes (8–10). Ectopic expression of FOXP3 in CD4^+^CD25^−^ T cells may endow CD4^+^CD25^−^ T cells with Treg-like suppressive capability to prevent inflammatory bowel disease (IBD) and autoimmune gastritis (9). FOXP3-deficient Treg cells have decreased levels of Treg cell signature genes, including *ctla4*, *ebi3*, *il10*, and *entpd1*, and acquire the expression of T effector cytokine genes such as *ifng, tnf*α*, il4, and il17* (11–14). A frame-shift mutation in the *foxp3* gene locus in scurfy mice results in the expression of FOXP3 protein lacking its forkhead domain (15). Many other loss-of-function mutations at the *foxp3* gene locus have also been identified in patients with immune-dysregulation, polyendocrinopathy, enteropathy, and X-linked inheritance syndrome (IPEX) (16, 17). Genetic mutations of the *foxp3* gene are always accompanied by the lack of the functional Treg cells, therefore resulting in the development of diverse arrays of autoimmune diseases. A compilation of studies describing the role of genetic mutants of the *foxp3* gene in autoimmune diseases is shown in Table 1.

**Table 1:** **The polymorphism of the *foxp3* gene in autoimmune diseases**.

Diseases	Polymorphisms and mutations	Location	Reference
Rheumatoid arthritis	(GT)n	Promoter	(18)
Systemic lupus erythematosus	rs3060515 rs3761548 (GT)n	Promoter Promoter Promoter	(19) (19) (18)
IPEX	rs6609857 (A-G) ΔE201 (A-G) A1087G G13128A	Intron Intron Exon Poly A region Exon Exon	(20) (17) (17) (21) (22) (23)
Type 1 diabetes	(GT)n rs4824747 (TC)n	Promoter Intron Intron	(24–26) (27) (24, 25, 28)

## FOXP3 and Treg Cell Development

**Figure 1:**
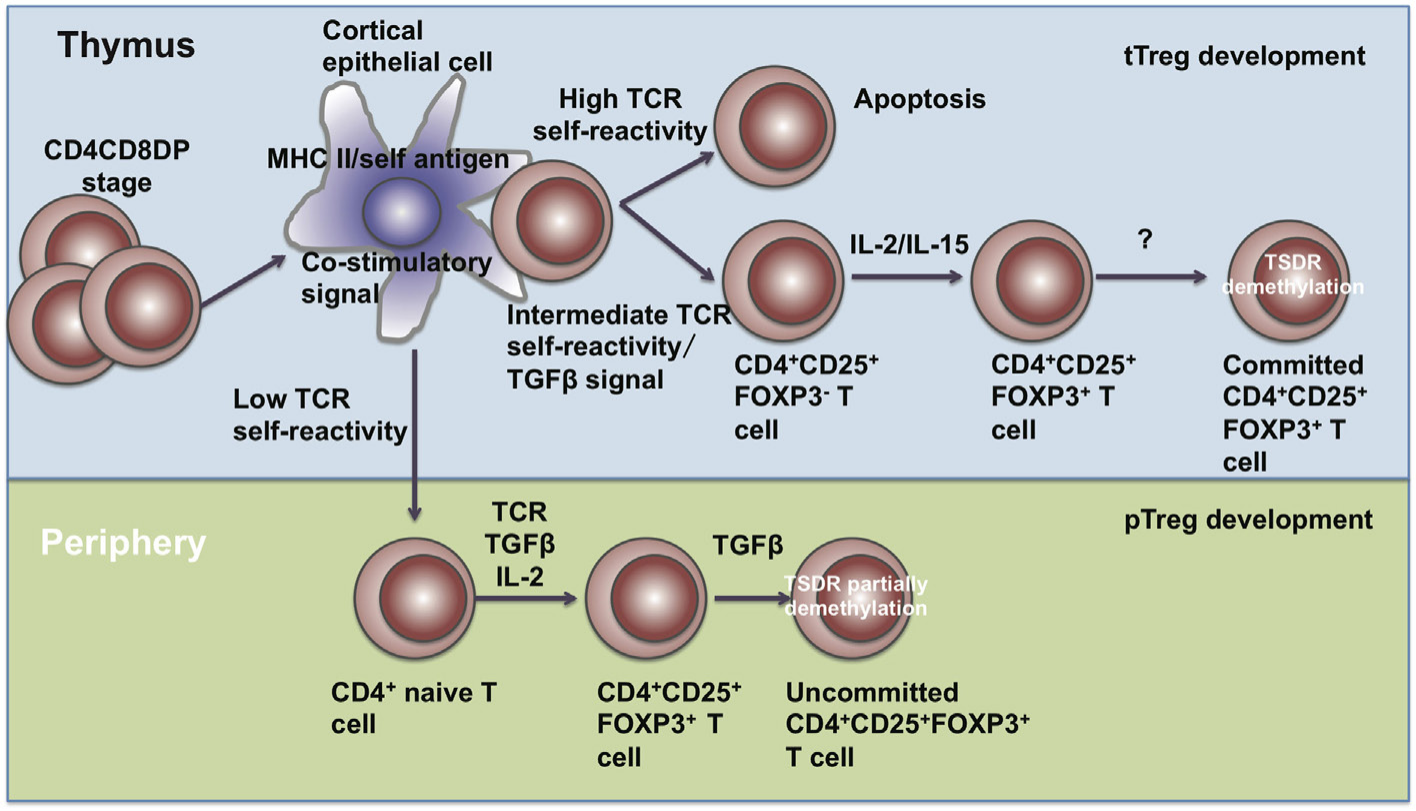
**The development of Treg cells**. Treg cells develop in the thymus and periphery. In the thymus, CD4^+^CD8^+^ T cells undergo negative selection and become mature tTreg cells through IL-2, IL-15, and TGF-β signals. In the periphery, naïve CD4^+^ T cells encounter antigen and differentiate into pTreg cells in the presence of TGF-β and IL-2.

Treg cells comprise approximately 5–15% of the CD4^+^ T cell compartment and can be subdivided into two subpopulations, including thymus-derived Treg (tTreg) cells and peripherally derived Treg (pTreg) cells. tTreg (also called natural Treg (nTreg)) cells are generated from Treg precursors at the immature HSA^hi^ CD4SP stage when FOXP3 is induced and Treg lineage commitment established (29). pTreg cells are differentiated from naïve T cells at peripheral sites in the presence of IL-2 and TGF-β (Figure 1). Those generated *in vitro* through TGF-β signals are known as induced Treg (iTreg) cells (30).

In the thymus, the development of tTreg cells requires extracellular signals, including TCR-mediated self-antigen recognition, γ chain cytokines, and TGF-β etc. DO11.10 transgenic mice expressing transgenic TCRs specific for an OVA peptide had normal proportions of CD4^+^CD8^−^CD25^+^ thymocytes, while DO11.10 transgenic mice with a RAG-2 gene-deficient background had fewer CD4^+^CD8^−^CD25^+^ thymocytes (31), suggesting that TCR signaling is required for the development of tTreg cells. Also, transgenic mice harboring T cells specific for the major I-E^d^ determinant (S1) of influenza hemagglutinin (HA) exhibited higher percentages and numbers of FOXP3^+^ Treg cells recognizing HA (32, 33), showing that the TCRs of tTreg are biased toward self-antigens. Intermediate TCR strength has also been reported to be required for tTreg development. Sequencing of TCRs has showed that Treg cells share little similarity with naïve T cells. The diversity of TCRs on Treg cells surpasses the diversity of TCRs from naïve T cells (34). Although some studies have reported no substantial differences between the TCR repertories of Treg and non-Treg cells, their conclusions may only be based on the usage of the TCR variable region segments Vβ or Vα and size distribution of complementarity-determining region 3 (CDR3) (35, 36). These parameters are too limited to determine the identity of individual TCRs and reflect the differences only when a clonotypic, oligoclonal response occurs. CD4^+^CD25^−^ T cells harboring the TCRα chains from Treg cells have been shown to expand faster when transferred into a lymphopenic host, suggesting that TCRs on Treg cells possess substantially higher affinity with MHC class II-bound self-peptides (37). In Nur77^GFP^ mice, the mean fluorescence intensity (MFI) of GFP revealed that the TCR signal strength in tTreg and pTreg cells was almost two-fold compared with conventional CD4^+^ T cells (38). All these studies indicate that Treg cells are self-reactive.

Besides TCR signaling, γ chain cytokines are also required for FOXP3 expression, including IL-2, IL-7, and IL-15. Treg cells express high levels of the IL-2 receptor α chain (CD25) (39). *il2rα*- or *il2rβ*-deficient mice have decreased numbers of Treg cells in spleens and lymph nodes and develop autoimmunity around 4–8 weeks of age (40–46). Other non-IL-2 cytokines through γc partially compensate for IL-2 signaling. In *il2*^−/−^ mice, CD4^+^FOXP3^+^ T cells were still detectable, but drastically reduced in *il2*^−^*^/^*^−^*il7*^−^*^/^*^−^, *il2*^−/−^*il15*^−/−^, *il2r*β^−/−^ and γ_c_^−/−^ mice (47). In the thymus, TGF-β signals prevent tTreg cell apoptosis. Conditional deletion of the TGF-β type I receptor (*Tgfbr1*) gene in T cells causes tTreg cells in the thymus to become more susceptible to apoptosis during negative selection, while bim ablation may restore TGF-β signal deficiency (48).

Recent studies showed that FOXP3 expression alone was not sufficient for Treg lineage commitment. The demethylation status of a Treg-specific demethylation region (TSDR) in the *foxp3* promoter plays an essential role in Treg lineage maintenance where the demethylation of the TSDR correlates with stable Treg cell phenotype. Gene expression profile analysis in FOXP3-non-expressing T cells that lacked methylation of the TSDR, and FOXP3-expressing T cells that retained methylation of the TSDR, showed higher similarity to tTreg cells in the former in gene expression but lack of repression in the expression of *il2, ifng, and zap70*; however, the latter cells exhibited normal *il2, ifng, and zap70* repression but upregulated a set of genes that were not expressed in tTreg cells. These results indicated that FOXP3 expression and the demethylation of the TSDR are both vital to establish Treg lineage commitment, but neither of them alone is sufficient (49).

In the periphery, combined TCR, TGF-β, and IL-2 signals polarize naïve CD4^+^ T cells into pTreg cells. These pTreg cells possess similar suppressive capacities as tTreg cells *in vitro* and *in vivo* (50, 51). Both tTreg and pTreg cells express FOXP3, CD25, CTLA-4, GITR, CD39, and CD73, along with low levels of IL-7Rα (CD127) (52). Current studies indicate that tTreg and pTreg cells play differential roles in different inflamed tissues. pTreg cells are more functional for maintaining mucosal tolerance, while tTreg cells are for maintaining immune tolerance. Due to the lack of specific lineage markers to distinguish between tTreg and pTreg cells in humans, it remains difficult to illustrate the different functions of tTreg and pTreg cells. Helios has been identified as a marker for tTreg cells (53). However, tTreg subsets have been found to contain both FOXP3^+^Helios^+^ and FOXP3^+^Helios^−^ subpopulations, suggesting that Helios is not a specific marker for tTreg/pTreg cells (54). Other studies have identified Neuropilin 1 (NRP1) specifically and highly expressed on tTreg cells but not pTreg cells (55), and glycoprotein A repetitions predominant (GARP) expressed on activated human tTreg cells but not TGFβ-induced iTreg cells (56), but subsequent reports found that NRP1^low^ pTreg cells could be converted into NRP1^hi^ pTreg cells under inflammatory environments (57). Therefore, other surface markers need to be discovered for distinguishing between tTreg and pTreg cells.

## The Stability of Treg Cells

As Treg cells have been identified as a specific cell population possessing suppressive capacity to maintain immune homeostasis, Treg cell therapy is seen as a promising method for treating autoimmune diseases. However, clinical trials for autoimmune disease indications thus far, via re-administration of expanded Treg cells into patients, have been far from satisfactory (58) as the phenotype and function of Treg cells may change *in vivo*. This raises the question of whether or not Treg cells are stable (59). Due to the ambiguity of specific Treg cell markers, FOXP3 is so far the most distinct marker to distinguish Treg cells from other T effector cells; therefore, most of the work aimed at elucidating the stability of Treg cells has been based on the expression of FOXP3.

Some investigations have shown that Treg cells are unstable and phenotypically flexible under certain inflammatory microenvironments, supported by evidence of how CD4^+^FOXP3^+^ Treg cells convert into T-helper-like cells with appropriate stimulation, including Th1-, Th2-, Th17-, and Tfh-like cells (60–63). Through adoptive transfer of CD4^+^EGFP^+^ and CD4^+^EGFP^−^ T cells from the spleen and LN of *Foxp3*^EGFP^ mice into *rag2*^−/−^ mice, investigators found that over 90% of the transferred eGFP^+^ T cells maintained FOXP3 expression, and a minor fraction lost their FOXP3 expression. Analysis of the minor fraction of T cells identified a population limited to the FOXP3^+^CD25^−^ subset that exhibits flexible responses to other cytokines, indicating that natural FOXP3^+^ T cells contained a committed Treg cell lineage and an uncommitted minor population (64).

Zhou et al. generated *Foxp3*–GFP–Cre × R26-YFP mice to track Foxp3^+^ T cells *in vivo* by crossing transgenic mice expressing a green fluorescent protein–Cre recombinase fusion protein (GFP–Cre) controlled by the *foxp3* promoter on a bacterial artificial chromosome (BAC; *Foxp3*–GFP–Cre mice) with reporter mice that express yellow fluorescent protein (YFP) driven by the *Rosa26* promoter only after excision of a *lox*P-flanked stop cassette (R26-YFP mice). YFP^+^GFP^−^ T cells represented cells that had expressed FOXP3 at some point before loss of expression, while YFP^+^GFP^+^ T cells represented stable FOXP3-expressing cells. They found approximately 15% of the YFP^+^ cells lost FOXP3 expression, and coined these as “exFoxp3 cells.” Characteristic analysis found that these exFoxp3 cells exhibited an activated-memory T cell phenotype and expressed inflammatory cytokines. Adoptive transfer of these cells *in vivo* caused rapid onset of diabetes (65).

Meanwhile, other researchers have shown that Treg cells are very stable, and suggest that the unstable Treg cells that have been observed are not bona fide Treg cells but an uncommitted “pre”-Treg cell lineage. To avoid the occurrence of monitoring transiently expressed FOXP3 in effector T cells, Rubtsov et al. generated *Foxp3*^GFP–Cre–ERT2^ ROSA26^YFP^ mice to distinguish cells that had only begun to express FOXP3 from those that expressed FOXP3 for a longer duration by detecting YFP intensity, and observed that only 3% of YFP^+^ cells had lost FOXP3 (66). Hori et al. carried out similar experiments with *Foxp3*^GFP–Cre^*ROSA26*^RFP^ knock-in mice, and claimed that exFOXP3 T cells were generated from transiently induced FOXP3^+^ T cells in lymphopenic environments but not from committed Treg cells (67).

## The Regulation of FOXP3 Expression

**Figure 2:**
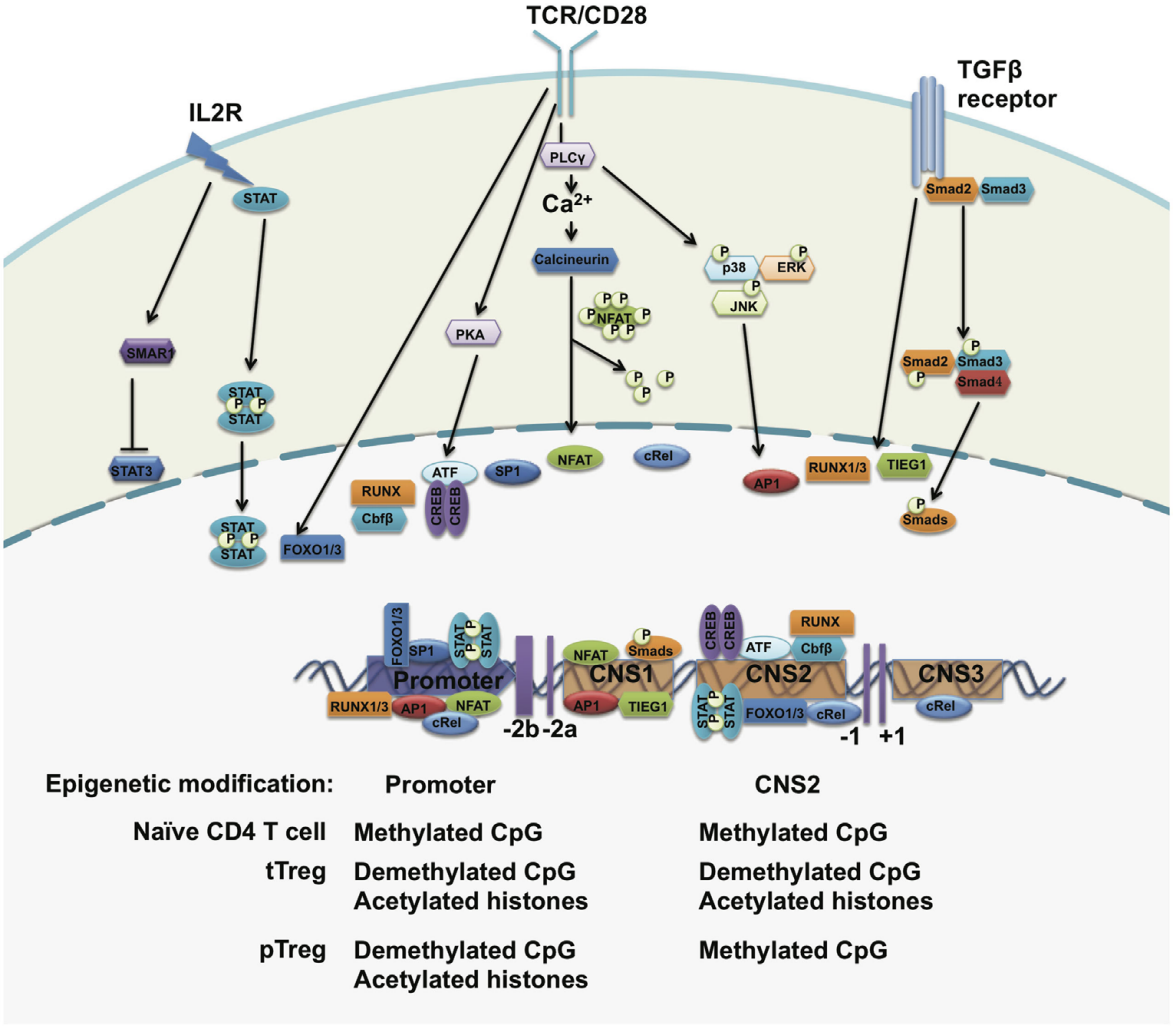
**The regulation of FOXP3 expression**. The *foxp3* promoter, three conserved regulatory regions, and the epigenetic modification status of the *foxp3* gene. Upon TCR stimulation, NFAT, AP1, Sp1, and CREB-ATF bind to the promoter of the *foxp3* gene. STAT5 forms a dimer in response to IL-2 signals and translocates to the *foxp3* promoter. In the periphery, TGF-β signals drive SMADs and NFAT occupancy at the CNS2 region and may induce FOXP3 expression. The CpG island within the *foxp3* promoter region is demethylated in both tTreg cells and pTreg cells but not in naïve T cells. The histones bound to the *foxp3* promoter region are hyperacetylated in both tTreg and pTreg cells. However, CNS2 is demethylated only in tTreg but not in pTreg cells.

The significance of FOXP3 to Treg development and stability is well documented. Direct evidence that has shown FOXP3 protein to be important for Treg function has been provided by experiments that inserted a gene cassette co-expressing luciferase and enhanced green fluorescent protein (eGFP) into the 3′-untranslated region (UTR) of the endogenous *foxp3* locus of C57BL/6 mice. This lead to FOXP3 mRNA instability, a 90% decrease of FOXP3 protein expression, and as a consequence these mice succumbed to aggressive lymphoproliferative autoimmune syndrome, indicating that Treg cell function directly correlates with the amount of FOXP3 protein expressed (12). Observations like this make it imperative to explore the molecular mechanisms regulating FOXP3 expression (Figure 2).

### Epigenetic control of FOXP3 expression

Epigenetic modifications of the *foxp3* gene at its regulatory regions regulate chromatin accessibility for transcription factors and other transcriptional regulators to control FOXP3 expression and Treg cell stability. Chromatin immunoprecipitation (ChIP) assays have revealed higher levels of acetylated histone H4 within the *foxp3* promoter in activated Treg cells (68). Treatment with histone deacetylase inhibitors leads to an increased expression of FOXP3 and percentages of FOXP3^+^ Treg cells *in vivo* (69), implying that the upregulation of FOXP3 expression is controlled by histone modifications. Both H3K4me2 and H3K4me3 are induced at the transcriptional start sites and regulatory regions at the *foxp3* gene locus in both tTreg and iTreg cells upon TCR stimulation (70). Inhibition of H3K4me3 at the *foxp3* gene locus impairs TGFβ-induced FOXP3 expression (71).

The methylation status of CpG islands within the *foxp3* promoter and regulatory elements also regulates the expression of FOXP3 in Treg cells. Through bisulfite sequencing, investigators have identified a CpG-rich region upstream of exon-1 of the *foxp3* gene locus and this region is highly conserved between human and mice. This evolutionarily conserved region is highly demethylated in tTreg cells, incompletely demethylated in iTreg cells, and methylated in naïve CD4^+^CD25^−^ T cells. This demethylated region is correlated with stable FOXP3 expression and closely associated with modified histones, including acetylated and trimethylated histone H3 but not acetylated histone H4 (72). Genome-wide DNA methylation pattern analysis confirmed specific CpG methylation patterns at other Treg cell-associated gene regions, including *il2ra*, *ctla4*, *tnfrsf18*, *ikzf4*, and *ikzf2* (49). Inhibition of DNA methylation by 5-aza-2′-deoxycytidine or deleting DNA methyltransferase-1 (DNMT-1) induces strong and stable expression of FOXP3 under TCR stimulation even in the absence of TGF-β, which further confirms that the TSDR methylation status of the *foxp3* gene locus controls the expression of FOXP3 (73, 74).

### Transcriptional regulation of FOXP3

Upon TCR activation, AP1, CREB, NFAT, c-Rel and ATF bind to the promoter of the *foxp3* gene and activate its gene transcription in Treg cells (68, 75–80). Foxo-binding sites were also found within the *foxp3* basal promoter, where deficiency of Foxo1 and Foxo3 in Treg cells causes a loss of FOXP3 expression (81). IL-2 signaling is essential to maintain FOXP3 expression in a STAT5-dependent manner (47, 82, 83). Additionally, IL-2 may induce the expression of SMAR1 in Treg cells, while IL-6 does the opposite. SMAR1-bound STAT3 promoters can suppress its gene transcription. Deficiency of SMAR1 in Treg cells causes the upregulation of STAT3, which in turn converts Tregs into Th17-like cells and facilitates increased susceptibility to IBD (84).

In the periphery, naïve T cells can be converted into FOXP3^+^ Treg cells in the presence of TGF-β. TGF-β induces the occupancy of Runx1 and Runx3 on the promoter of *foxp3*, but also activates SMAD3 and NFAT binding to the conserved non-coding sequence 1(CNS1) of the *foxp3* gene and induces FOXP3 expression (78, 85–90). Thus, CNS1 is considered to be involved in the development of pTreg cells in response to TGF-β signals. In CNS1-deficient mice, FOXP3^+^ Treg cells are markedly decreased in the gut-associated lymphoid tissue (GALT) and mesenteric lymph node (MLN), where TGF-β-dependent pTreg cells are generated, but not in the spleen and non-gut draining lymph nodes (91). In addition, RA was reported to be capable of augmenting the enrichment of SMADs to CNS1 and therefore enhances FOXP3 expression (88).

Conserved non-coding sequence 2(CNS2) was identified as a unique region containing CpG-rich islands to maintain stable FOXP3 expression in mature tTreg cells. In naïve T cells and pTreg cells, CNS2 is hypermethylated by DNMT-1 and occupied by HDACs and Mecp2 to repress the expression of FOXP3. Under the stimulation of TCR signals plus IL-2, DNMT-1 is released from CNS2 and induces demethylation (47, 77, 83, 92). The transcription factors CREB, STAT5, Est1, c-Rel, FOXP3, Runx–Cbfb heterodimer, and Foxo1/3 are recruited to this element to initiate FOXP3 transcription (77, 81, 86, 91, 93, 94). Deletion of CNS2 induces a loss of FOXP3 protein in mature Treg cells in the presence of IL-6, IFNγ, IL-12, and IL-4 (95, 96). However, a high amount of IL-2 rescues the loss of FOXP3 expression through enhancing STAT5 enrichment onto the *foxp3* basal promoter (73, 74).

Conserved non-coding sequence 3(CNS3) is also responsible for the induction of FOXP3. Conditional knockouts of CNS3 in Treg cells can markedly decrease the frequency of tTreg cells and may impair TGF-β-mediated pTreg induction (91). c-Rel was found to bind to this region to drive FOXP3 expression (91).

## The FOXP3 Protein Complex and Its Modifications

FOXP3 cooperates with various cofactors to induce the Treg cell gene expression signature and tailor their suppressive function. Biochemical and mass-spectrometric studies showed that FOXP3 could associate with several hundred partners to form a large multi-protein complex (97, 98). FOXP3 cooperates with NFAT and AML1/Runx1 to regulate the expression of IL-2, CD25, and CTLA4 through binding to their promoters and activating gene transcription. Disruption of their interaction would impair Treg suppressive function (99, 100). The association of FOXP3 with Eos–CtBP co-repressor complexes is required for FOXP3-mediated IL-2 repression in Treg cells. In a colitis mouse model, Eos-deficient Treg cells failed to repress the development of adoptive colitis (101). Additionally, a FOXP3–IRF4 complex contributes to establishing Treg-specific gene programs. A conditional knockout of IRF4 in Treg cells showed elevated Th2 responses (102). Deleted in breast cancer 1 (DBC1), a subunit of the FOXP3 complex, prevents FOXP3 degradation and maintains Treg cell stability under inflammatory conditions. Functional Dbc1^−/−^ mice are more resistant to develop severe autoimmune disease symptoms during induction of experimental autoimmune encephalomyelitis (EAE) (103).

The transcription factor GATA3 is highly induced in Treg cells that reside in barrier sites, including the gastrointestinal tract and skin. GATA3 is required for maintaining high levels of FOXP3 expression by binding to and promoting the activity of cis-acting elements of FOXP3. GATA3-deficient Treg cells are more prone to acquire an effector T cell phenotype and express effector cytokines in inflamed tissues (104, 105). USP21 positively regulates and stabilizes GATA3, which can maintain FOXP3 expression. Furthermore, USP21-knockout mice show spontaneous T cell activation (106, 107). Tbet and RORgt have also been identified to be essential for Th1-like and Th17-like Treg cells in inflammatory microenvironments, respectively, and promote Treg cell homing to inflamed loci (108–110).

### Post-translational modifications of FOXP3

**Figure 3:**
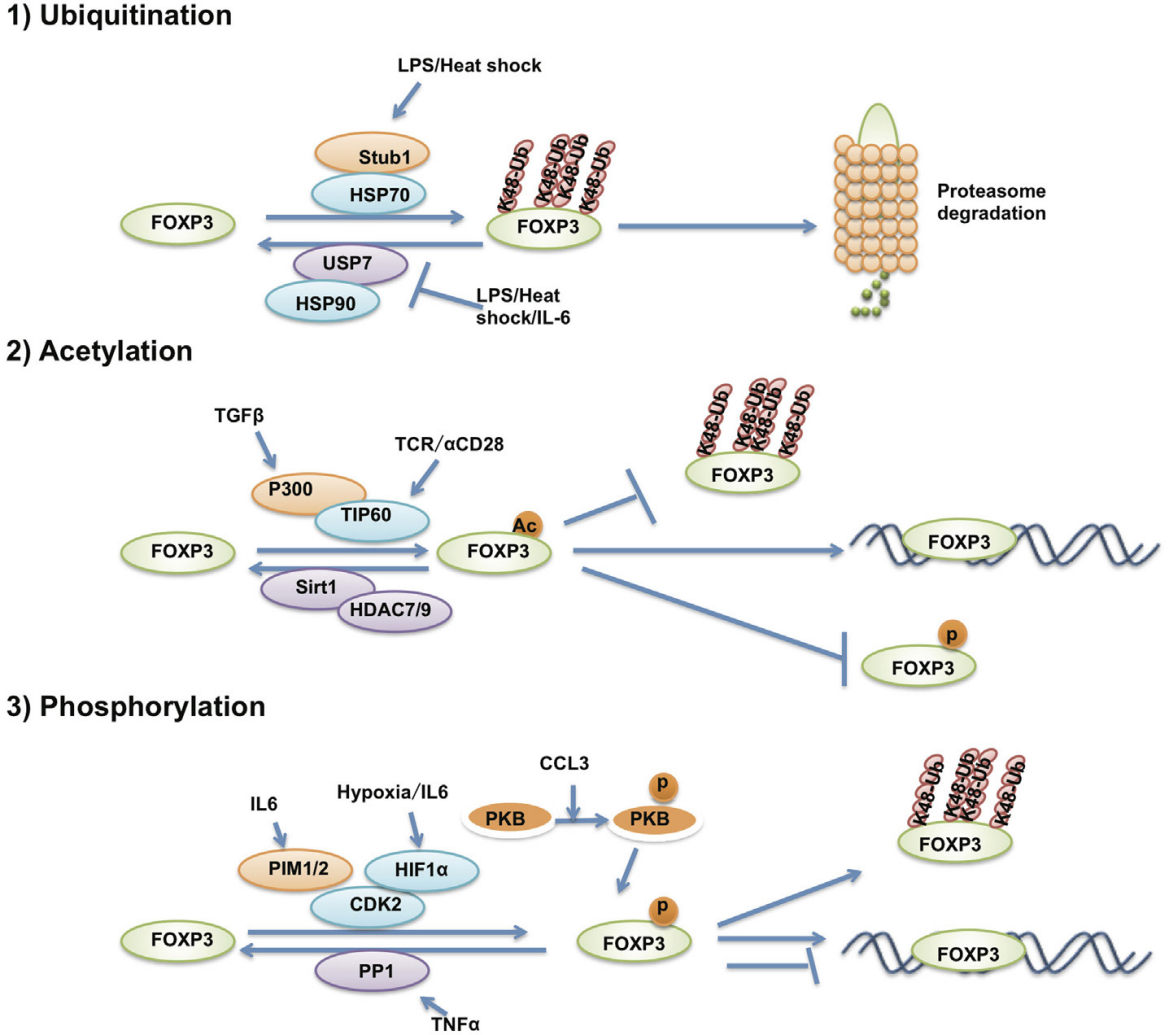
**The post-translational modification of FOXP3**. The post-translational modifications that affect FOXP3 stability and transcriptional activity. FOXP3 protein is ubiquitinated, acetylated and phosphorylated by various post-translational modification enzymes.

The post-translational modifications of FOXP3 affect Treg differentiation, function, and phenotypic commitment through regulating FOXP3 protein stability and transcriptional activity (Figure 3). Several previous studies have reported that FOXP3 protein stability is controlled by ubiquitination-mediated degradation. Under inflammatory conditions, STUB1 was found recruited to FOXP3 by HSP70 to polyubiquitinate FOXP3 at its K227/250/263/268 sites in a K48-linked polyubiquitination manner. K48-linked polyubiquitinated FOXP3 is further led to proteasome-mediated degradation. Manipulating the level of STUB1 in Treg cells through ectopic expression or knockdown directly affected the protein levels of FOXP3, signature Treg gene expression and the ability to suppress inflammatory immune responses (111). On the other hand, the deubiquitinase USP7 is able to deubiquitinate FOXP3 in an HSP90-dependent manner and stabilizes FOXP3 to increase Treg number to enhance Treg suppressive activity (112). HIF1a and PKB/Akt1-mediated FOXP3 phosphorylation also affects FOXP3 stabilization through indirectly regulating FOXP3 ubiquitination levels (113–116).

The transcriptional activity of FOXP3 is also regulated by post-translational modifications. Our previous results demonstrated that FOXP3 could associate with the histone acetyltransferase TIP60 and the class II histone deacetylases HDAC9 and HDAC7. TIP60 can acetylate FOXP3 and enhance FOXP3-mediated transcription repression of IL-2 expression through the FOXP3 N-terminal 106 to 109aa region (117). FOXP3 can also be acetylated by P300 and affects FOXP3 stability through impairing polyubiquitination of FOXP3, thus, blocking proteasome-mediated FOXP3 degradation (114, 115).

In addition to ubiquitination and acetylation, MS analysis has revealed that multiple residues of FOXP3 could be phosphorylated. Among these, only a small number have been further investigated. In the synovial fluid of rheumatoid arthritis patients, the pro-inflammatory cytokine TNFα induces the expression and enzymatic activation of protein phosphatase 1 (PP1) that dephosphorylates Ser418 of FOXP3. Subsequently, FOXP3 loses its transcription repression of IL-2 and Treg cells lose their suppressive function, causing increased numbers of IL-17^+^ and IFN-γ^+^CD4^+^ T cells within the inflamed synovium of rheumatoid arthritis patients (118). IL-6-induced PIM1 can phosphorylate Ser422 of FOXP3, which negatively regulates FOXP3 binding affinity on chromatin and also Treg function. Reversing PIM1-mediated FOXP3 phosphorylation through TCR stimulation, shRNA-mediated PIM1 depletion or by using a PIM1 inhibitor could enhance Treg suppressive function (119). Another member of the PIM kinase family named PIM2 was also reported to be able to phosphorylate multiple sites of FOXP3 at its N-terminal domain, leading to attenuated Treg suppressive function. *Pim2*^−/−^ mice show more resistance to DSS-induced colitis (117). FOXP3 is also the target of CDK2, which phosphorylates FOXP3 at its Ser19 and Thr175 sites to negatively regulate the stability and transcriptional activity of FOXP3 (120). Although most investigations have reported that FOXP3 is strictly expressed in Treg cells, FOXP3 can also be expressed in cancer cells and acts as a cancer repressor (121, 122). Lck can also phosphorylate FOXP3 at Tyr342 in breast cancer cells and increase FOXP3 transcriptional repression of *mmp9*, *skp*, and *vegfa*, and thus suppresses cellular invasion (123).

## Treg Cells and Gender Bias in Autoimmune Diseases

Females and males process basic immune responses rather differently. In response to infection, vaccination, or trauma, females exhibit stronger inflammation for protection against infection, while this characteristic also renders females more susceptible to autoimmune diseases. The factors that contribute to these disparate immune responses between males and females are mainly X-linked, which includes hormonal differences.

Current theories related to the pathogenesis of autoimmune diseases assume that the disrupted balance between effector T cells (that cause tissue damage) and Treg cells (that suppress self-reactive cells) correlates with the pathogenesis of autoimmune diseases. The number and function of Treg cells is affected by X-linked *foxp3* and hormonal fluctuations. Thus, new insight into gender differences in autoimmune disease may reveal novel therapeutic avenues.

### Treg and IPEX

The *foxp3* gene is localized on the X chromosome, where mutations in this gene may cause IPEX. In females, there are two X chromosomes, where one undergoes random inactivation. If the *foxp3* gene on one X chromosome is mutated, this would potentially produce functionally impaired Treg cells, whereas the other gene with the wild-type *foxp3* gene would generate normal Treg cells to protect females from IPEX (124).

### Treg cells and MS

Multiple sclerosis is characterized by chronic inflammation, primary demyelination, and axonal damage. EAE is the animal model of MS. In adoptive transfer experiments, Treg cells may prevent the development of chronic EAE in recipient mice (125–127), implying that Treg cells contribute to protection against MS. Investigators have found no differences in the frequency of CD4^+^CD25^hi^ Treg cells between patients with MS and healthy controls, while several groups revealed how CD4^+^CD25^hi^ Treg cells in MS patients are functionally impaired (128–131). MS is more prevalent in females (132). In females, the symptoms of MS have been reported to correlate with hormonal levels. When estrogen (E2) and progesterone (P4) levels decrease during menstruation, disease relapses (133, 134); in turn, during the third trimester of pregnancy when estrogen and progesterone levels are at its highest, the symptoms of MS regress, followed by relapse until dropping at post-partum (135, 136). Treatment with ER ligand protected mice from the development of EAE (137, 138). The protective effect of ER ligand was blocked in estrogen receptor-α (Esr1^−/−^)- and estrogen receptor-β (Esr2^−/−^)-deficient mice (138). Both E2 and P4 have been reported to induce high numbers of Treg cells and enhance Treg function (139–142). E2 treatment increased Treg cell number and FOXP3 expression both *in vitro* and *in vivo*. In estrogen receptor-α-deficient mice, E2-induced expression of FOXP3 is abrogated (141, 143). E2 was reported to regulate Treg function partially through increasing intracellular levels of the checkpoint inhibitor PD-1. PD-1 expression and Treg suppressive function were attenuated in ER-KO mice. E2 pre-treatment could partially restore the suppressive function of Treg cells in PD-1 KO mice without affecting FOXP3 expression (144).

Other reports have revealed how 17β-estradiol enhances Treg suppressive function via promoting TGF-β and IL-10 secretion (145). P4 may drive cord blood fetal T cells but not adult peripheral blood T cells to differentiate into FOXP3^+^ Treg cells. These P4-induced Treg cells exhibit a memory phenotype and better suppressive activity. Mechanistically, P4 enhances IL-2-STAT5 signaling and represses IL-6-mediated STAT3 activation by downregulating the IL-6 receptor, facilitating Treg differentiation but suppression of Th17 differentiation (139). P4 could also suppress the mTOR pathway, and thus promote the generation of Treg cells (146) and these Treg express higher levels of ERβ compared with T-responder cells. In MS patients, Treg cells express lower levels of ERβ (147), thus implying that having Treg cells unresponsive to hormones might result in the dysregulation of immune homeostasis and contribute to the pathogenesis of MS.

Frequencies of Treg cells change during the course of pregnancy (148). During pregnancy, elevated E2 levels at early stages are important for CD4^+^CD25^+^ Treg cell expansion in mice and are required for embryo implantation (149). Estrogen-treated mice and pregnant mice share similarities in increases of FOXP3 expression and Treg function (150). E2 and P4 increase maintains the expansion of systemic and local uterine Treg cells (140). The correlation between pregnancy-induced fluctuations in Treg cells and MS amelioration remain unclear, which might be influenced by different flow-cytometric approaches and current lack of studies.

### Treg cells and SLE

The imbalance of Th17/Treg cells usually correlates with the pathogenesis of SLE (151, 152). For SLE, data have shown a gender bias toward prevalence in females, with the female:male ratio at almost 9:1 (132). IL-6 plays a very important role in regulating the balance between Th17 cells and Treg cells. In the presence of IL-6, naïve CD4^+^ T cells differentiate into Th17 cells (with TGF-β) rather than iTreg cells (153). IL-6 together with IL-1 induces the degradation of FOXP3 and deregulates Treg cells (61). Higher concentrations of IL-6 in sera and in urine have been detected in SLE patients; the concentration of IL-6 in SLE patient sera and urine is positively correlated with disease severity (154–157). The expression of IL-6 is upregulated by estrogens (158) and is dominant in females (159). In mice, blocking IL-6 could significantly increase FOXP3 expression and make animals resistant to ALD-DNA-induced SLE (160). IL-6 may affect Th17/Treg balance in males and females, and thus contributes to the prevalence of SLE in females. So far, related studies are limited and more evidence is required to further characterize this correlation.

### Treg cells and AS

Ankylosing spondylitis (AS) is a chronic inflammatory disease with strong genetic connections (161, 162). Patients with AS are two to three times higher in males than females, and suffer from inflammatory spinal pain that could lead to the pathogenesis of spondyloarthritis and spinal immobility (163). Treatment of AS by tumor necrosis factor α inhibitors seem effective, which leads to the reduction of disease progression (164). The imbalance of Treg cells and inflammatory Th17 cells in AS patients has been previously studied but the underlying mechanism remains unclear (165, 166). Small molecule inhibitors that promote Treg function could play a beneficial role in preventing the pathogenesis of AS (167, 168).

## Conclusion

Accumulating experimental evidence has revealed the important role of Treg cells in maintaining immune homeostasis and preventing the occurrence of autoimmune diseases. Treg cells adopt multiple molecular mechanisms to maintain their lineage stability and obtain a certain degree of functional plasticity to adapt to various inflammatory conditions. However, inflammatory factors from the local microenvironment would interfere with the stability of Treg cells and promote the development of autoimmune diseases. Therefore, exploring the molecular mechanisms behind the function of the Treg cell-lineage transcription factor FOXP3 in autoimmunity would provide insight into the understanding of the stability and plasticity of Treg cells. Treg therapy could be an important tool for treating autoimmune disease in the future. Current reports describing the effect of gender differences on Treg cells and the contributions of Treg cells to the prevalence of autoimmune diseases in females are limited. The latest findings that Treg cells are regulated by hormonal fluctuations suggest that these risk factors that may disrupt the balance between T helper and Treg cells and induce autoimmune disease include birth control pills, stress, existence or development of ovarian cysts, and overuse of products containing xenoestrogens, etc., causing hormonal imbalance. Hence, it is significantly important to take sex-based differences into consideration when exploring the role of Treg cells in human illnesses and development of Treg cell therapies for treating autoimmune diseases.

Although Treg cells are well acknowledged as a potential and promising tool for the treatment of autoimmune diseases, there is still a large gap between theory and reality. To achieve the goal of successfully and effectively using Treg cells to restore tolerance and for treating autoimmune diseases, the following important questions in Treg cell biology still need to be further addressed:

1. Except for NRP1, Helios, and GARP, are there better surface makers for distinguishing between tTreg and pTreg cells, and what are the different physiological functions of tTreg and pTreg cells in the context of autoimmune disease?
2. How is the FOXP3 complex and post-translational modifications dynamically regulated in response to various physiological signals and how do they modify Treg cell function?
3. What is the role of Treg cells in the onset and progression of different autoimmune diseases?
4. What is the correlation between Treg cells and gender bias in different autoimmune diseases?

## Conflict of Interest Statement

The authors declare that the research was conducted in the absence of any commercial or financial relationships that could be construed as a potential conflict of interest.
